# Adaptive penalization in high-dimensional regression and classification with external covariates using variational Bayes

**DOI:** 10.1093/biostatistics/kxz034

**Published:** 2019-10-09

**Authors:** Britta Velten, Wolfgang Huber

**Affiliations:** Genome Biology Unit, European Molecular Biology Laboratory, Meyerhofstr. 1, 69117 Heidelberg, Germany

**Keywords:** Classification, External covariates, Feature selection, Penalized regression, Variational Bayes

## Abstract

Penalization schemes like Lasso or ridge regression are routinely used to regress a response of interest on a high-dimensional set of potential predictors. Despite being decisive, the question of the relative strength of penalization is often glossed over and only implicitly determined by the scale of individual predictors. At the same time, additional information on the predictors is available in many applications but left unused. Here, we propose to make use of such external covariates to adapt the penalization in a data-driven manner. We present a method that differentially penalizes feature groups defined by the covariates and adapts the relative strength of penalization to the information content of each group. Using techniques from the Bayesian tool-set our procedure combines shrinkage with feature selection and provides a scalable optimization scheme. We demonstrate in simulations that the method accurately recovers the true effect sizes and sparsity patterns per feature group. Furthermore, it leads to an improved prediction performance in situations where the groups have strong differences in dynamic range. In applications to data from high-throughput biology, the method enables re-weighting the importance of feature groups from different assays. Overall, using available covariates extends the range of applications of penalized regression, improves model interpretability and can improve prediction performance.

## 1. Introduction

We are interested in the setup where we observe a continuous or categorical response $Y$ together with a vector of potential predictors, or features, $X \in \mathbb{R}^p$ and aim to find a relationship of the form $ Y = f(X).$ Two main questions are of potential interest in this setting. First, we want to obtain an $f$ that yields good predictions for $Y$ given a new observation $X$. Second, we aim at finding which components in $X$ are the “important ones” for the prediction.

A common and useful approach to this end are (generalized) linear regression methods, which assume that the distribution of $Y|X$ depends on $X$ via a linear term $X^T\beta$. In order to cope with high-dimensionality of $X$ and avoid over-fitting, penalization on $\beta$ is employed, e.g., in ridge regression (Hoerl and Kennard, 1970), Lasso (Tibshirani, 1996), or elastic net (Zou and Hastie, 2005). By constraining the values of $\beta$, the complexity of the model is restricted, resulting in biased but less variable estimates and improved prediction performance. In addition, some choices of the penalty yield estimates with a relatively small number of non-zero components, thereby facilitating feature selection. An example is the $L_1$-penalty employed in Lasso or elastic net.

Commonly, penalization methods apply a penalty that is symmetric in the model coefficients. Real data, however, often consist of a collection of heterogeneous features, which such an approach does not account for. In particular, it ignores any additional information or structural differences that may be present in the features. Often we encounter $X$ whose components comprise multiple data modalities and data qualities, e.g., measurement values from different assays. Other side-information on individual features could include temporal or spatial information, quality metrics associated to each measurement or the features’ sample variance, frequency or signal-to-noise ratio. It has already been observed in multiple testing that the power of the analysis can be improved by making use of such external information (e.g., Ferkingstad *and others*, 2008; Dobriban *and others*, 2015; Ignatiadis *and others*, 2016; Li and Barber, 2019; Lei and Fithian, 2018). However, in current penalized regression models this information is frequently ignored. Making use of it could on one hand improve prediction performance. On the other hand, it might yield important insight into the relationship of external covariates to the features’ importance. For example, if the covariate encodes different data modalities, insights into their relative importance could help cutting costs by reducing future assays to the essential data modalities.

As a motivating example, we consider applications in molecular biology and precision medicine. Here, the aim is to predict phenotypic outcomes, such as treatment response, and identify reliable disease markers based on molecular data. Nowadays, different high-throughput technologies can be combined to jointly measure thousands of molecular features from different biological layers (Ritchie *and others*, 2015; Hasin *and others*, 2017). Examples include genetic alterations, gene expression, methylation patterns, protein abundances, or microbiome occurrences. However, despite the increasing availability of molecular and clinical data, outcome prediction remains challenging (Hamburg and Collins, 2010; Chen and Snyder, 2013; Alyass *and others*, 2015). Common applications of penalized regression only make use of parts of the available data. For example, different assay types are simply concatenated or analyzed separately. In addition, available annotations on individual features are left unused, such as their chromosomal location or gene set and pathway membership. Incorporating side-information on the assay type and spatial or functional annotations could help to improve prediction performance. Furthermore, it could help prioritizing feature groups, such as different assays or gene sets.

Here, we propose a method that incorporates external covariates in order to guide penalization and can learn relationships of the covariate to the feature’s effect size in a data-driven way. We introduce the method for linear models and extend it to classification purposes. We demonstrate that this can improve prediction performance and yields insights into the relative importance of different feature sets, both on simulated data and applications in high-throughput biology.

## 2. Methods

### 2.1. Problem statement

Assume we are given observations $(x_1, y_1), \dots, (x_n, y_n)$ with $y_i \in \mathcal{Y} \subseteq \mathbb{R}$, $x_i \in  \mathbb{R}^p$ (possibly $n\ll p$) from a linear model, i.e.

(2.1) \begin{equation*} y_i = x_i^T\beta +\epsilon_i \end{equation*}

with $\epsilon_i \stackrel{iid}{\sim} \text{N}(0, \sigma^2)$. In addition, we suppose that we have access to a covariate $\zeta_j \in \mathcal{Z} \subseteq \mathbb{R}^k$ for each predictor $j=1,\dots, p$. We hope, loosely speaking, that $\zeta_j$ contains some sort of information on the magnitude of $\beta_j$. The question we want to address is: can we use the information from $\zeta$ to improve upon estimation of $\beta$ and prediction of $Y$?

In order to estimate $\beta$ from a finite sample $y=(y_i)_{i=1,\dots,n} \in \mathbb{R}^{n}$ and $\mathbf{X} = [x_1,\dots, x_n]^T \in \mathbb{R}^{n \times p}$ we can employ penalization on the negative log-likelihood of the model, i.e.

(2.2) \begin{equation*} \hat{\beta}(\lambda) \in \arg \min_\beta \frac{1}{n}||y - \mathbf{X}\beta||_2^2 + \lambda p(\beta), \label{eq:pen} \end{equation*}

where $p$ denotes a penalty function on the model coefficients. For example, $p(\beta) = \sum_j |\beta_j|^q$ leads to Lasso ($q=1$) or ridge regression ($q=2$). The parameter $\lambda$ controls the amount of penalization and thereby the model complexity. Ideally, we would like to choose an optimal $\lambda$. For estimation this means minimizing the mean squared error $\text{MSE}(\hat{\beta}(\lambda)) = \mathbb{E} ||\hat{\beta}(\lambda) - \beta ||_2^2$; for prediction this means minimizing the expected prediction error. In practice, $\lambda$ is often chosen to minimize the cross-validated error.

In most applications, the penalization is symmetric, i.e., for any permutation $\pi$ we have $\lambda p(\beta_1,\dots, \beta_p) = \lambda p(\beta_{\pi(1)}, \dots, \beta_{\pi(p)})$. However, as we have external information on each feature given by $\zeta$ we want to allow for differential penalization guided by $\zeta$. For this, we will consider the following non-symmetric generalization, which still leads to a convex optimization problem in $\beta$ for convex penalty functions $\tilde{p}$, such as $\tilde{p}(x) = |x|$ or $\tilde{p}(x) = x^2$:

(2.3) \begin{equation*} \hat{\beta}(\lambda) \in \arg \min_\beta \frac{1}{n}||{y} - \mathbf{X}\beta||_2^2 + \sum_j \lambda(\zeta_j) \tilde{p}(\beta_j). \label{eq:diffPen} \end{equation*}

Instead of a constant $\lambda$, here $\lambda\,{:}\,\mathcal{Z} \rightarrow \mathbb{R}_{\geq0}$ provides a mapping from the covariate $\zeta$ to a non-negative penalty factor $\lambda(\zeta)$. This additional flexibility compared to a single penalty parameter can be helpful if $\zeta$ contains information on $\beta$. For example, in the simple case of ridge regression with deterministic orthonormal design matrix, known noise variance $\sigma^2$ and “oracle covariate” $\zeta_j = \beta_j$ the optimal $\lambda$ is seen to be $\lambda^*(\zeta_j) = \frac{\sigma^2}{\zeta_j^2}$. However, in practice the information in $\zeta$ is not that explicit and hence we do not know which $\lambda$ is optimal.

If $\lambda$ takes values in a small set of discrete values, e.g., for categorical covariates $\zeta$, cross-validation could be used to determine a suitable set of function values. This approach is employed by Boulesteix *and others* (2017), where categorical covariates encode different data modalities. However, cross-validation soon becomes prohibitive, as it requires a grid search exponential in the number of categories defined by $\zeta$. Similarly, cross-validation can be employed with $\lambda$ parametrized by a small number of tuning parameters using domain knowledge to come up with a suitable parametric form for $\lambda$ (Bergersen *and others*, 2011; Veríssimo *and others*, 2016). However, such an explicit form is often not available. In many situations, it is a major problem itself to come up with a helpful relationship between $\zeta$ and $\beta$ and thereby knowledge of which values of a covariate would require more or less penalization. Therefore, we aim at finding $\lambda$ in a data-driven manner and with improved scalability compared to cross-validation.

### 2.2. Problem statement from a Bayesian perspective

There is a direct correspondence between estimates obtained from penalized regression and a Bayesian estimate with penalization via corresponding priors on the coefficients. For example, the ridge estimate corresponds to the maximum a posterior estimate (MAP) in a Bayesian regression model with normal prior on $\beta$ and the Lasso estimate to a MAP with a Laplace prior on $\beta$. This correspondence opens up alternative strategies using tools from the Bayesian mindset to approach the problem outlined above: Differential penalization translates to introducing different priors on the components of $\beta$. Our belief that $\zeta$ carries information on $\beta$ can be incorporated by using prior distributions whose parameters depend on $\zeta$. Wiel *and others* (2016) used this idea to derive an Empirical Bayes approach for finding group-wise penalty parameters in ridge regression. However, this approach does not obviously generalize to other penalties such as the Lasso.

Moving completely into the Bayesian mindset we instead turn to explicit specification of priors to implement the penalization task. Different priors have been suggested (Mitchell and Beauchamp, 1988; MacKay, 1996; Park and Casella, 2008; Carvalho *and others*, 2009) and structural knowledge was incorporated into the penalization by employing multivariate priors that encode the structure in the covariance or non-exchangeable priors with different hyper-parameters (e.g., Hernández-Lobato *and others*, 2013; Engelhardt and Adams, 2014;Rockova and Lesaffre, 2014; Wu *and others*, 2014; Andersen *and others*, 2017; Xu and Ghosh, 2015 and references therein). Despite the possible gains in prediction performance when incorporating such structural knowledge, these methods have not been widely applied. A limiting factor has often been the lack of scalability to large datasets.

### 2.3. Setup and notation

From the linear model assumption we have

(2.4) \begin{equation*} y_i=x_i^T\beta +\epsilon_i \qquad \epsilon_i \stackrel{iid}{\sim} \text{N}(0,{\tau}^{-1}), \end{equation*}

where $\tau$ denotes the precision of the noise. Based on the external covariate $\zeta$ we define a partition of the $p$ predictors into $G$ groups:

(2.5) \begin{equation*} g_\zeta = g:\{1,\dots, p\} \rightarrow \{1,\dots, G\}. \end{equation*}

For instance, categorical covariates $\zeta$, such as different assay types, naturally define such a partition. For continuous covariates $g_\zeta$ can be defined based on suitable binning or clustering.

To achieve penalization in dependence of $\zeta$ we consider a spike-and-slab prior (Mitchell and Beauchamp, 1988) on the model coefficients $\beta$ with a different slab precision $\gamma$ and mixing parameter $\pi$ for each group. We re-parametrize $\beta$ as $\beta_j=s_j b_j$ with

(2.6) \begin{align*} b_j|\gamma_{g_\zeta(j)} &\sim \text{N}\left(0,\gamma_{g_\zeta(j)}^{-1}\right)\!, \label{ss-normal}\\ \end{align*}

(2.7) \begin{align*} s_j|\pi_{g_\zeta(j)}&\sim \text{Ber}(\pi_{g_\zeta(j)}). \label{ss-ber} \end{align*}

In the special case of $\pi=1$, this yields a normal prior as in MacKay (1996) corresponding to ridge regression. With $\pi<1$ we additionally promote sparsity on the coefficients, and the value of $\pi$ controls the number of active predictors in each group. The value of $\gamma$ controls the overall shrinkage per group. To learn the model hyper-parameters $\gamma$, $\pi$ and the noise precision $\tau$, we choose the following conjugate priors

(2.8) \begin{align*} \tau &\sim\Gamma(r_\tau, d_\tau),\\ \end{align*}

and for each group $k \in \{1,\dots, G\}$

(2.9) \begin{align*} \gamma_k &\sim \Gamma(r_\gamma, d_\gamma),\\ \end{align*}

(2.10) \begin{align*} \pi_k &\sim \operatorname{Beta}(d_\pi, r_\pi), \end{align*}

with $d_\tau,r_\tau, d_\gamma , r_\gamma = 0.001 $ and $r_\pi, d_\pi = 1$. Hence, the joint probability of the model is given by

(2.11) \begin{align*} p(y,b,s,\gamma,\pi,\tau)=p(y|b,s, \tau) p(b,s|\pi,\gamma)p(\gamma)p(\pi)p(\tau). \label{eq:model} \end{align*}

### 2.4. Inference using variational Bayes

The challenge now lies in inferring the posterior of the model parameters from the observed data ${\mathbf X},y$ and the covariate $\zeta$. While Markov Chain Monte Carlo methods are frequently used for this purpose they do not scale well to large datasets. Here, we adopt a variational inference framework (Bishop, 2006; Blei *and others*, 2017) that has been used (in combination with importance sampling) for variable selection with exchangeable priors (Carbonetto and Stephens, 2012; Carbonetto *and others*, 2017). Denoting all unobserved model components by $\theta =(b,s, \gamma, \pi, \tau)$, we approximate the posterior $p(\theta|{\mathbf X},y)$ by a distribution $q(\theta)$ from a restricted class of distributions $\mathcal{Q}$, where the goodness of the approximation is measured in terms of the Kullback–Leibler (KL) divergence, i.e.

(2.12) \begin{equation*} q \in \arg\min_{q \in \mathcal{Q}} D_{\text{KL}}(q\,||\,p(\theta|{\mathbf X},y)). \end{equation*}

A common and useful choice for distributions in class $\mathcal{Q}$ is the mean-field approximation, i.e., that the distribution factorizes in its parameters. We consider

(2.13) \begin{align*} q(\theta) = q(b,s,\gamma, \pi, \tau)= \prod_{j=1}^p q(b_j,s_j)q(\gamma)q(\pi)q(\tau), \label{eq:MFA} \end{align*}

where $b_j$ and $s_j$ are not factorized due to their strong dependencies (Titsias and Lázaro-Gredilla, 2011).

The variational approach leads to an iterative inference algorithm (Blei *and others*, 2017) by observing that minimizing the KL-divergence is equivalent to maximizing the evidence lower bound $\mathcal{L}$ defined by

(2.14) \begin{align*} \log(p(y))=\mathcal{L}(q)+D_{\text{KL}}(q \,||\,p(\theta\,|\,{\mathbf X},y)). \label{eq:ELBOdef} \end{align*}

From this, we have

(2.15) \begin{align*} \mathcal{L}(q)&= \int \log \frac{p(y, \theta)}{q(\theta)}\, q(\theta) \,d\theta\\ \end{align*}

(2.16) \begin{align*} &= \int \log p(y, \theta)\, q(\theta)\, d\theta +H(q(\theta)), \end{align*}

with $H(q)=\int - q(\theta)\, \log q(\theta) \, d\theta$ denoting the differential entropy.

Variational methods are based on maximization of the functional $\mathcal{L}$ with respect to $q$ in order to obtain a tight lower bound on the log model evidence and minimize the KL-divergence between the density $q$ and the true (intractable) posterior. Under a mean-field assumption $q(\theta) = \prod_j q(\theta_j)$, the optimal $q_j$ keeping all other factors fixed is given by

(2.17) \begin{equation*} \log(q_j^*)(\theta_j)= \mathbb{E}_{-j}(\log(p(y,\theta))) -\text{const}. \end{equation*}

Iterative optimization of each factor results in Algorithm S1 of the supplementary material available at *Biostatistics* online. Further details on the variational inference and the updates can be found in Sections 1 and 2 of the supplementary material available at *Biostatistics* online. The method is implemented in the open-source Bioconductor package *graper*. From the obtained approximation $q$ of the posterior distribution, we obtain point estimates for the model parameters. In particular, we will use the posterior means $\hat{\beta} = \int \beta \, q(\beta) \, {\rm d}\beta$, $\hat{\gamma} = \int \gamma \, q(\gamma)\, {\rm d}\gamma$, and $\hat{\pi} = \int \pi\, q(\pi) \,{\rm d}\pi$.

**Remark on the choice of the mean-field assumption**.

An interesting deviation from the standard fully factorized mean-field assumption in Equation (2.13) is taking a multivariate variational distribution for the model coefficients. This is easily possible for the dense model ($\pi=1, s=1, b = \beta$), where we can consider the factorization

$$        \begin{align*}
        q(\beta, \gamma, \tau)=q(\beta)q(\gamma)q(\tau).
        \end{align*}$$

In particular, a multivariate distribution is kept for the model coefficients $\beta$ instead of factorizing $q(\beta) = \prod_j q(\beta_j)$. Thereby, this approach allows to capture dependencies between model coefficients in the inferred posterior and is less approximative. We will show below that this can improve the prediction results. However, a drawback of this approach is its computational complexity, as it requires the calculation and inversion of a $p\times p$ covariance matrix in each step. While this can be reduced to a quadratic complexity as described in Section 2.1 of the supplementary material available at *Biostatistics* online, this is still prohibitive for many applications. Therefore, we concentrate in the following on the fully factorized mean-field assumption but include comparisons to the multivariate approach in the Results.

### 2.5. Extension to logistic regression

The model of Section 2.3 can be flexibly adapted to other types of generalized linear regression setups with suitable link functions and likelihoods. However, the inference framework needs to be adapted due to loss of conjugacy. Here, we extend the model to the framework of logistic regression with a binary response variable, where we assume that the response follows a Bernoulli likelihood with a logistic link function

(2.18) \begin{align*} y_i|\beta \sim \text{Ber}(\sigma(x_i^T\beta)) \quad \text{with} \quad \sigma(z)=\frac{1}{1+\exp(-z)}. \end{align*}

While the prior structure and core of the variational inference are identical to the case of a linear model, additional approximations are necessary. For this purpose, we adopt work by Jaakkola and Jordan (2000) and approximate the likelihood using a lower bound on the logistic function. For an arbitrary $\xi \in \mathbb{R}$ we have

(2.19) \begin{equation*} \sigma(z)\geq \sigma(\xi)\exp\left(\frac{1}{2}(z-\xi)-\eta(\xi)(z^2-\xi^2)\right) \end{equation*}

with $\eta(\xi)=\frac{1}{2\xi}\left(\sigma(\xi)-\frac{1}{2}\right)$. With this, $\log p(y|\beta)=\sum_{i=1}^n \log(\sigma((2y_i - 1) x_i^T\beta))$ can be bounded by

(2.20) \begin{align*} \begin{split} \log p(y|\beta) &\geq \frac{1}{2}\sum_i (2y_i-1) x_i^T \beta -\sum_i \eta(\xi_i) (x_i^T \beta)^2 \\ & \qquad+\sum_i \left( \log(\sigma(\xi_i))-\frac{1}{2}\xi_i +\eta(\xi_i) \xi_i^2 \right)\!. \end{split} \end{align*}

As this approximation restores a quadratic form in $\beta$, the remaining updates can be adopted from the case of a linear model above with the additional variational parameter $\xi$ (see Section 2.2 of the supplementary material available at *Biostatistics* online for details).

## 3. Results

### 3.1. Results on simulated data

First, we evaluated the method on simulated data to test its ability to recover the model coefficients and hyper-parameters per group. For this, a random ${\mathbf X}$ matrix was generated from a multivariate normal distribution with mean zero and a Toeplitz covariance structure $\Sigma_{ij}=\rho^{|i-j|}$, and the response was simulated from a linear model with normal error. The $p$ predictors were split into $G=6$ groups of equal size, and the coefficients were simulated from the model as described in Equations (2.6) and (2.7) with fixed $\pi_k$ and $\gamma_k$ for each group. In particular, we set $\gamma_k =0.01$ for $k=1,2$, $\gamma_k =1$ for $k=3,4$, and $\gamma_k =100$ for $k=5,6$. For each pair of groups with same $\gamma$-value the sparsity level $\pi_k$ was varied between $\nu$ and $\min(1,1.5\nu)$ for a certain value of $\nu$ determining the sparsity level from 0 (sparse) to 1 (dense). We then varied the number of features $p$, the number of samples $n$, the correlation strength $\rho$, the noise precision $\tau$, and the sparsity level $\nu$ (Table S1 of the supplementary material available at *Biostatistics* online) and generated for each setting ten independent datasets. We evaluated the recovery of the hyper-parameter $\gamma$ and $\pi$ for each group and compared the predictive performance and computational complexity to those of related methods including ridge regression (Hoerl and Kennard, 1970), Lasso (Tibshirani, 1996), elastic net (Zou and Hastie, 2005), adaptive Lasso (Zou, 2006), sparse group Lasso, group Lasso (Friedman *and others*, 2010), GRridge (Wiel *and others*, 2016), varbvs (Carbonetto *and others*, 2017), and IPF-Lasso (Boulesteix *and others*, 2017). Those methods were taken from the respective R packages provided by the authors, i.e., *glmnet 2.0-16, SGL 1.1, grpreg 3.2-0, GRridge 1.7.1, varbvs 2.4-0, and ipflasso 0.1*.

#### 3.1.1. Recovery of hyper-parameters

The algorithm accurately recovered the relative importance of different groups (encoded by $\gamma_k$) and the group-wise sparsity level (encoded by $\pi_k$) across a large range of settings as shown in Figure S1 of the supplementary material available at *Biostatistics* online. The method failed to recover those parameters accurately only if the ratio between sample size and number of features was too small or the sparsity parameter $\nu$ was too close to 1. These settings were challenging for all methods as can be seen in Section 3.1.2, where we evaluated estimation and prediction performance in comparison to other methods. In addition, the groups had to contain sufficiently many predictors to reliably estimate group-wise parameters, as seen in Figure S1b of the supplementary material available at *Biostatistics* online. We also noted that a low signal-to-noise ratio could impede the estimation of hyper-parameters as can be seen from the group with a very large $\gamma$ value (meaning low coefficient amplitudes as in group 5 and 6) and low precision values ($\tau$) of the noise term.

#### 3.1.2. Prediction and estimation performance

**Fig. 1.**
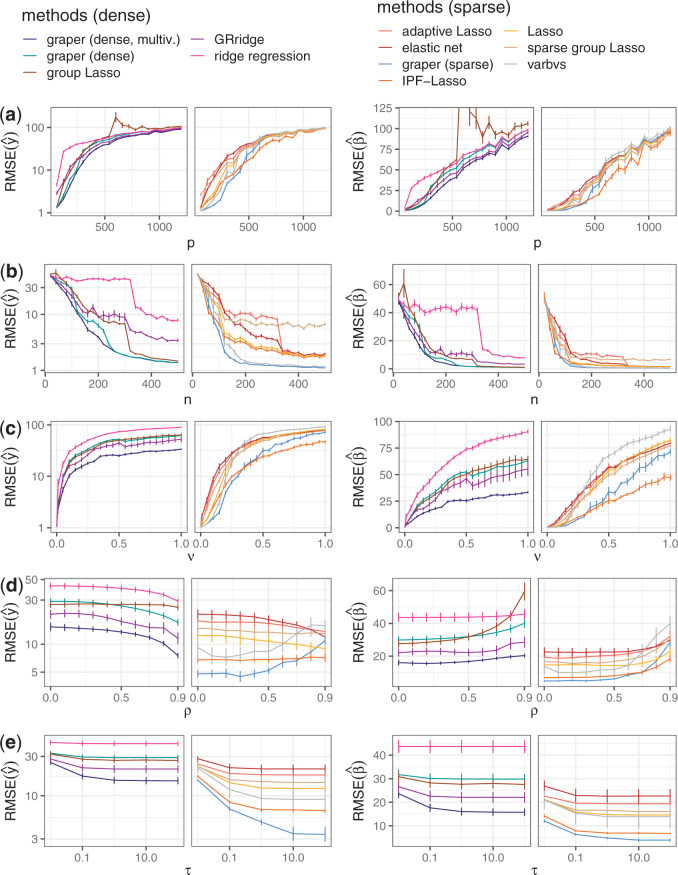
Root mean squared error (RMSE) of the predicted response $\hat{y} = X^T\hat{\beta}$ (left) and the estimate $\hat{\beta}$ (right) for different methods when varying one of the simulation parameters (a–e) as described in Table S1 of supplementary material available at *Biostatistics* online. The prediction error is assessed on $n=1000$ test samples. The line denotes the mean RMSE across 10 random instances of simulated data with bars denoting standard errors. The two panels separate methods with sparse estimates of $\beta$ (right) from non-sparse methods (left). (Group Lasso is counted as non-sparse method as it is not sparse within groups.)

Next, we compared the estimation of the true model coefficients and the prediction accuracy on an independent test set of $n=1000$. Overall, the method showed improved performance for a large range of sample sizes, correlations, numbers of features, noise variances and active features, both in terms of the root mean squared error on $y$ as well as for estimation of $\beta$ (Figure 1). Among the non-sparse methods, graper with a non-factorized mean-field assumption clearly outperformed the factorized mean-field assumption as well as GRridge and group Lasso. The covariate-agnostic ridge regression performed worst in most cases. Sparse methods performed in general better in this simulation example, as the underlying model had a large fraction of zero coefficients. Here, we observed that graper was comparable to IPF-Lasso, which is the most closely related method. Only in settings with a very high number of active predictors or strong correlations between the predictors ($\rho$ close to one) the method was outperformed by the IPF-Lasso.

#### 3.1.3. Scalability

**Fig. 2.**
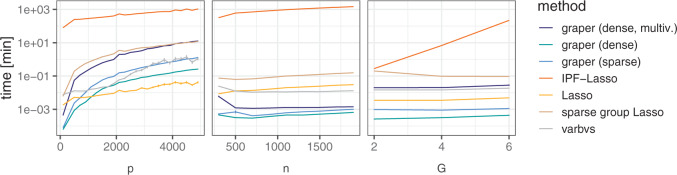
Average run time (in min) for different methods when varying the number of samples $n$, features $p$ and groups $G$. Each parameter is varied at a time while holding the others fixed to $n=100$, $p=300$ or $G=6$. Shown are the average times across 50 random instances of simulated data with error bars denoting one standard error.

While the additional group-wise optimization comes at a computational cost, the variational approach runs inference in time complexity linear in the number of features $p$, samples $n$, and groups $G$. Only in the case of a multivariate variational distribution, the complexity is quadratic in the larger of $n$ and $p$ and cubic in the smaller of the two. When varying the number of samples $n$, features $p$, and groups $G$ we observed comparable run times as for Lasso (Figure 2). Differences were mainly observed for $p$: For larger $p$, graper required slightly longer times than Lasso. This difference was more pronounced when using a sparsity promoting spike and slab prior, where additional parameters need to be inferred. As expected, the multivariate approach of graper became considerably slower for large $p$ and showed comparable run times to the sparse group Lasso. The number of groups mainly influenced the computation times of IPF-Lasso, which scales exponentially in the number of groups. Here, graper provided a by far more scalable approach (Figure 2, right panel).

### 3.2. Application to data from high-throughput biology

#### 3.2.1. Drug response prediction in leukemia samples

Next, we exemplify the method’s performance on real data by considering an application to biological data, where predictors were obtained from different assays. Using assay type as external covariates we used the method to integrate data from the different assays (also referred to as omic types) in a study on chronic lymphocytic leukemia (Dietrich *and others*, 2018). This study combined drug response measurements with molecular profiling including gene expression and methylation. Briefly, we used normalized RNA-Seq expression values of the 5000 most variable genes, the DNA methylation M-values at the 1$\%$ most variable CpG sites as well as the ex-vivo cell viability after exposure to 61 drugs at five different concentrations as predictors for the response to a drug (Ibrutinib) that was not included into the set of predictors. The data were obtained from the Bioconductor package *MOFAdata 1.0.0* (Argelaguet *and others*, 2018). In total, this resulted in a model with $n=121$ patient samples and $p=9553$ predictors.

**Fig. 3.**
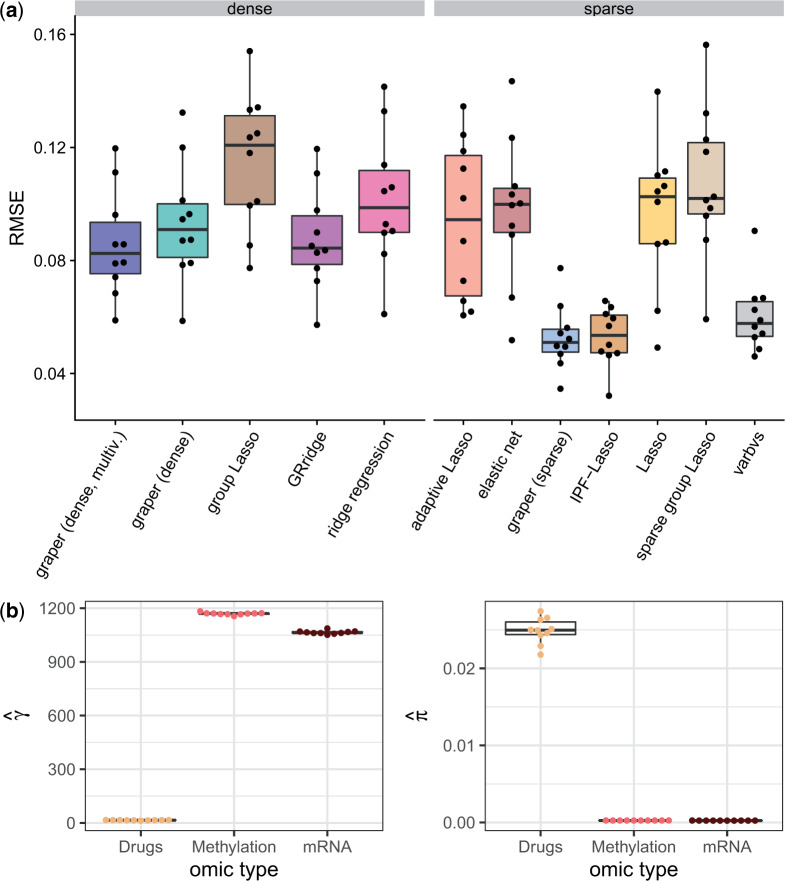
Application to the chronic lymphocytic leukemima data with scale differences between assays. (a) Comparison of the root mean squared error (RMSE) for the prediction of samples’ viability after treatment with Ibrutinib. Performance was evaluated in a 10-fold cross-validation scheme, the points denote the individual RMSE for each fold. (b) Inferred hyper-parameters in the different folds for the three different omic types ($\gamma$ on the left and $\pi$ on the right).

We first applied the different regression methods to the data on their original scale. Since the features have different scales (e.g., the drug responses vary from around 1 (neutral) to 0 (completely toxic), the normalized expression values from 0 to 20 and the methylation M-values from $-$10 to 8), this ensures that the omic type information is an informative covariate: it results in larger effect sizes of the drug response data and smaller effect sizes of the methylation and expression data compared to scaled predictors. In this setting, incorporating knowledge on the assay type into the penalized regression showed clear advantages in terms of prediction performance: The covariate-aware methods (GRridge, IPF-Lasso, and graper) all improved upon the covariate-agnostic Lasso, ridge regression, or elastic net (Figure 3a). Also the group Lasso methods, which incorporate the group information but apply a single penalty parameter, could not adapt to the scale differences. The inferred hyper-parameters $\gamma$ of graper highlighted the larger effect sizes of the drug response feature group, which was strongly favored by the penalization (Figure 3b).

**Fig. 4.**
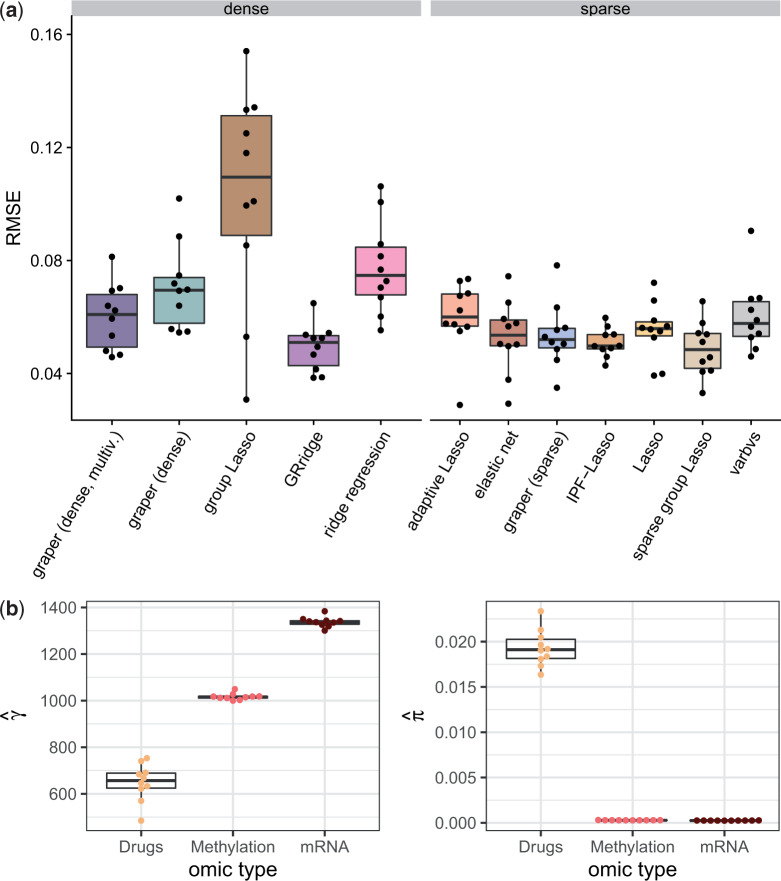
Application to the chronic lymphocytic leukemima data with standardized predictors. (a) Comparison of the root mean squared error (RMSE) for the prediction of samples’ viability after treatment with Ibrutinib. Performance was evaluated in a 10-fold cross-validation scheme, the points denote the individual RMSE for each fold. (b) Inferred hyper-parameters by graper (sparse) in the different folds for the three different omic types ($\gamma$ on the left and $\pi$ on the right).

To address differences in feature scale, a common choice made by many implementations (e.g., glmnet (Friedman *and others*, 2010)) is to scale all features to unit variance. Indeed, for the data at hand, this transformation was particularly beneficial for the covariate-agnostic methods, and their prediction performances became more similar to those of the covariate-aware methods. However, for dense methods such as ridge regression the covariate information on the omic type remained important (Figure 4a). Sparse methods in general resulted in very good predictions as the response to Ibrutinib can be well explained by a very sparse model containing only few drugs with related mode of action. By learning weights for each omic type graper directly highlighted the importance of the drug data as predictors (Figure 4b).

In general, standardization of all features is unlikely to be an optimal choice, since in many applications there is a relation between information content and amplitude. For example, we often measure high-amplitude signals that are informative jointly with low-amplitude features that originate mainly from technical noise. Here, standardization can be harmful as it would drown the informative high-amplitude features and “blow up” the noisy low-amplitude features (Figure S2 of the supplementary material available at *Biostatistics* online). In particular, standardization does not distinguish between meaningful differences in variance (e.g., features that differ between two disease groups) and differences in variance due to the scale. While removal of the latter would be desirable, meaningful differences should be retained. Hence, the question of whether to scale the predictors or not, is related to the question of whether the variance of a feature is an informative covariate: if the variance contains important information on the relevance of a predictor, standardization should not be applied or information on the predictors’ variance should be re-included via the covariate, e.g., binning features based on their variance. This has been shown to be beneficial for marginal testing applications where filtering or weighting by variance increased the power to detect true positives (Bourgon *and others*, 2010; Ignatiadis *and others*, 2016). A recent study on RNA-Seq data in the context of penalized regression found no strong effect of standardization compared to no standardization (Zwiener *and others*, 2014). However, an example where standardization can be harmful in applications to genomic data are binary mutation data. Here, features are all on the same scale and standardization would favor mutations with lower frequencies which in most applications is not desirable (see also Section 3.1 of the supplementary material available at *Biostatistics* online).

#### 3.2.2. Age prediction from multi-tissue gene expression data

As a second example for a covariate in genomics, we considered the tissue type. Using data from the GTEx consortium (Lonsdale *and others*, 2013), we asked whether the tissue type is an informative covariate in the prediction of a person’s age from gene expression. Briefly, using gene expression data provided by the Bioconductor package *recount 1.7.6* (Collado-Torres *and others*, 2017), we chose five tissues that were available for the largest number of donors and from each tissue considered the top 50 principal components on the RNA-Seq data after normalization and variance stabilization using *DESeq2 1.21.25* (Love *and others*, 2014). In total, this gave us $p=250$ predictors from $G=5$ tissues for $n=251$ donors.

**Fig. 5.**
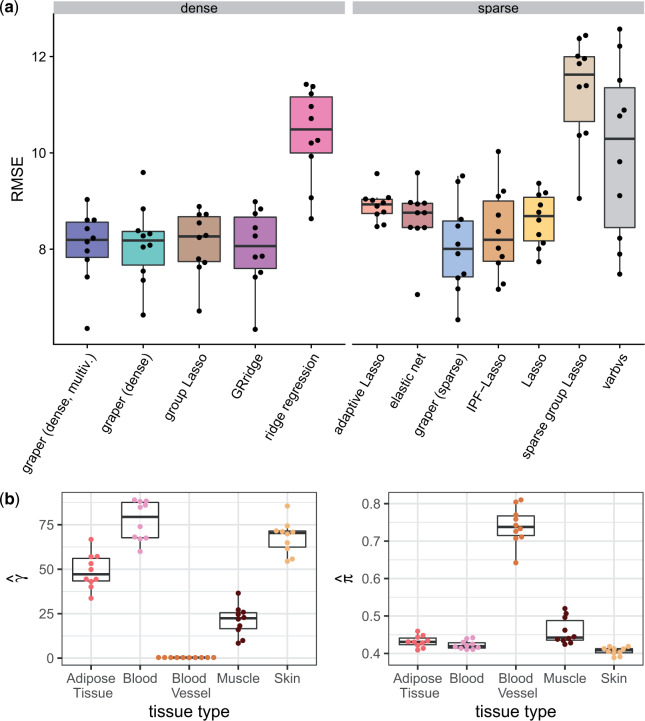
Application to the GTEx data. (a) Comparison of root mean squared error (RMSE) for the prediction of donor age (in years). Performance is evaluated in a 10-fold cross-validation scheme, the points denote the individual RMSE for each fold. (b) Inferred penalty parameters for the five tissues in graper in each fold.

We observed a small advantage for methods that incorporate the tissue type as a covariate (Figure 5a): GRridge, IPF-Lasso, and graper all had a smaller prediction error compared to covariate-agnostic methods. In particular, graper resulted in comparable prediction performance to IPF-Lasso, whilst requiring less than a second for training compared to 40 min for IPF-Lasso. The learnt relative penalization strength and sparsity levels of graper can again provide insights into the relative importance of the different tissue types. In particular, we found lower penalization for blood vessel and muscle and higher penalization for blood and skin (Figure 5b). This is consistent with previous studies on a per-tissue basis, where gene expression in blood vessel has been found to be a good predictor for age, while blood was found to be less predictive (Yang *and others*, 2015).

## 4. Discussion

We propose a method that can use information from external covariates to guide penalization in regression tasks and that can provide a flexible and scalable alternative to approaches that were proposed recently (Wiel *and others*, 2016; Boulesteix *and others*, 2017). We illustrated in simulations and data from biological applications that if the covariate is informative of the effect sizes in the model, these approaches can improve upon commonly used penalized regression methods that are agnostic to such information. We investigated the use of important covariates in genomics such as omic type or tissue. The performance of our approach is in many cases comparable to the IPF-Lasso method (Boulesteix *and others*, 2017), while scalability is highly improved in terms of the number of feature groups, thereby extending the range of possible applications.

The variational inference framework provides improved scalability compared to Bayesian methods that are based on sampling strategies. Variational Bayes methods have already been employed in the setting of Bayesian regression with Spike-and-Slab priors (Carbonetto and Stephens 2012; Carbonetto *and others* 2017). However, these methods do not incorporate information from external covariates. A drawback of variational methods is the fact that they often result in too concentrated approximations to the posterior distribution and thereby underestimate the posterior variance. Nevertheless, they have been shown to provide reasonable point estimates in regression tasks (Carbonetto and Stephens, 2012), which we focused on here. Due to the mean-field assumption strong correlations between active predictors can lead to suboptimal results of graper. Here, a multivariate mean-field assumption in the variational Bayes approach can be of advantage, suggested as an alternative above. However, it comes at the price of higher computational costs. What is not addressed in our current implementation is the common problem of missing values in the data; if extant, they would need to be imputed beforehand.

While our approach is related to methods that adapt the penalty function in order to incorporate structural knowledge, such as the group Lasso (Yuan and Lin, 2006), sparse group Lasso (Friedman *and others*, 2010), or fused Lasso ( Tibshirani *and others*, 2005), these approaches apply the same penalty parameter to all the different groups and perform hard in- or exclusion of groups instead of the softer weighting proposed here. Alternatively, the loss function can be modified to incorporate prior knowledge based on a known set of “high-confidence predictors” as proposed by Jiang *and others* (2016). The existence and identity of such “high-confidence predictors,” however, is often not clear.

In contrast to frequentist regression methods, the Bayesian approach provides direct posterior-inclusion probabilities for each feature that can be useful for model selection. To obtain frequentist guarantees on the selected features it could be promising to combine the approach with recently developed methods for controlling the false discovery rate, such as the knockoffs (Candes *and others*, 2018). For this, feature statistics can be constructed based on the estimated coefficients or inclusion probabilities from our model as long as the knockoffs obtain the same covariate information as their true counterpart.

An interesting question that we have not addressed is the quest for rigorous criteria when the inclusion of a covariate by differential penalization is of advantage. This question is not limited to the framework of penalized regression but affects the general setting of shrinkage estimation. While joint shrinkage of a set of estimates can be very powerful in producing more stable estimates with reduced variance, care needs to be taken on which measurements to combine in such a shrinkage approach. As in the case of coefficients in the linear model setting, external covariates could be helpful for this decision and facilitate a more informed shrinkage. However, allowing for differential shrinkage will reintroduce some degrees of freedom into the model and can only be advantageous if the covariate provides “sufficient” information to balance this. For future work, it would be of interest to find general conditions for when this is the case, thereby enabling an informed choice of covariates in practice.

We provide an open-source implementation of our method in the Bioconductor package *graper*. In addition, vignettes and scripts are made available that facilitate the comparison of graper with various related regression methods and can be used to reproduce all results contained in this work (https://git.embl.de/bvelten/graper_analyses).

## 5. Software

The method is implemented in the Bioconductor package *graper*, scripts for the analyses contained in this article can be found at https://git.embl.de/bvelten/graper_analyses.

## Supplementary Material

kxz034_Supplementary_Data
