# Supplementary material for: Adaptive penalization in high-dimensional regression and classification with external covariates using variational Bayes
Source: Biostatistics. 2019 Oct 9;22(2):348–64. doi: 10.1093/biostatistics/kxz034 (PMC8036004; doi:10.1093/biostatistics/kxz034)
Supplement: kxz034_Supplementary_Data [file kxz034_supplementary_data.zip › biosts-18300-File013.pdf]

Biostatistics (2019), **0**, 0, *pp.* 1–20
doi:10.1093/biostatistics/appendix

Adaptive penalization in high-dimensional regression and classification with external covariates using variational Bayes - Supplementary Materials

BRITTA VELTEN and WOLFGANG HUBER

European Molecular Biology Laboratory, Heidelberg, Germany

Here we provide details on the variational inference scheme, the updates in our model and practical considerations for training. As before, $\mathbf{X}$ denotes the $n \times p$ matrix of observed predictors and y the n-vector of observed response values. With $\mathbf{X}_{ij}$ we denote the (i, j)-th element of the matrix $\mathbf{X}$ and $\mathbf{X}_{\cdot,j}$ its j-th column. Furthermore, $y_i \in \mathbb{R}$ denotes the i-th response value and $x_i \in \mathbb{R}^p$ the i-th predictor vector, corresponding to the i-th row in $\mathbf{X}$. We will use $\mathbb{E} = \mathbb{E}_q$ to denote expectations with respect to the variational distribution q.

1. Variational inference

To arrive at a simple iterative algorithm, we make use of the following lemma, which provides an update rule for each factor in the variational distribution (Blei *and others*, 2017).

Lemma 1.1 Under the mean-field assumption and for a fixed j the evidence lower bound defined in Equation (2.14) is maximised by

$$\log(q_j^*(\theta_j)) = \mathbb{E}_{-j}(\log(p(y, \theta))) - \text{const},$$

© The Author 2019. Published by Oxford University Press. All rights reserved. For permissions, please e-mail: journals.permissions@oup.com

where the expectation is taken under the current variational distribution $\prod_{l \neq j} q(\theta_l)$.

This can be easily seen by writing

$$
\begin{aligned}
\mathcal{L}(q) &= \mathbb{E}_q \left(\log \frac{p(y,\theta)}{q(\theta)} \right) \\
&= \int q(\theta)(\log p(y,\theta) - \log q(\theta)) d\theta \\
&= \int q_j(\theta_j) \int (\log p(y,\beta,\gamma,\tau) - \log q(\theta_j)) \prod_{i \neq j} q(\theta_i) d\theta_{-j} d\theta_j \\
&\quad - \int \sum_{i \neq j} \log q(\theta_i) \prod_{i \neq j} q(\theta_i) \int q(\theta_j) d\theta_j d\theta_{-j} \\
&= \int q_j(\theta_j)(\mathbb{E}_{-j}(\log p(y,\theta)) - \log q(\theta_j)) d\theta_j - \text{const} \\
&= \int q_j(\theta_j) \log \left(\frac{\exp \mathbb{E}_{-j}(\log p(y,\theta))}{q(\theta_j)} \right) d\theta_j - \text{const} \\
&= -D_{\text{KL}}(q_j \,||\, \exp \mathbb{E}_{-j}(\log p(y,\theta))).
\end{aligned}
$$

Hence, after normalising, the distribution $q_i^*(\theta_i)$ is given by

$$
q_i^*(\theta_i) = \frac{\exp(\mathbb{E}_{-j}[\log p(y,\theta)])}{\int \exp(\mathbb{E}_{-j}[\log p(y,\theta)]) d\theta_j}.
$$

2. Update equations for the variational inference

2.1 *Linear regression model*

In the linear model we assume that the likelihood is given by

$$
y|\beta,\tau \sim \mathrm{N}\left(\mathbf{X}\beta, \frac{1}{\tau}\mathbb{1} \right).
$$

With the priors as described in Section 2.3 and again denoting $\beta = sb$ the joint distribution is given by

$$
p(y,b,s,\gamma,\pi,\tau) = p(y|b,s,\tau)p(b,s|\pi,\gamma)p(\gamma)p(\pi)p(\tau).
$$

Hence,

$$\log p(y, b, s, \gamma, \pi, \tau) = \text{const} + \frac{n}{2}\log(\tau) - \frac{\tau}{2}||y - \mathbf{X}(b \odot s)||_2^2$$
$$+ \sum_{j=1}^{p} \left\{\log(\pi_{g(j)})s_j + \log(1 - \pi_{g(j)})(1 - s_j)\right\}$$
$$+ \sum_{j=1}^{p} \left\{\frac{1}{2}\log(\gamma_{g(j)}) - \frac{\gamma_{g(j)}}{2} b_j^2\right\}$$
$$+ \sum_{k=1}^{G} \{(r_\gamma - 1)\log(\gamma_k) - d_\gamma \gamma_k\}$$
$$+ \sum_{k=1}^{G} \{(d_\pi - 1)\log(\pi_k) + (r_\pi - 1)\log(1 - \pi_k) - \log(B(d_\pi, r_\pi)\}$$
$$+ (r_\tau - 1)\log(\tau) - d_\tau \tau.$$

The dense model without the spike and slab component arises as a special case when dropping π and s from the model and setting $\beta = b$.

For the start we will make a full mean-field assumption, i.e.

$$q(b, s, \gamma, \pi, \tau) = \prod_{j=1}^{p} q(b_j, s_j)q(\gamma)q(\pi)q(\tau),$$

allowing only a joint distribution for (b_j, s_j) due to their strong dependencies (Titsias and Lázaro-Gredilla, 2011).

Denoting with θ all individual parameter components in the mean-field assumption, the updates are given by

$$\log(q_j(\theta_j)) = \mathbb{E}_{-j} \log(p(y, \theta)),$$

as shown above (Lemma 1.1). Thanks to conjugacy between the chosen priors and the likelihood these updates maintain the distributional family of θ_j reducing the inference to updates of their parameters in each step. The resulting algorithm can be found in Algorithm S1. In the following, we provide details on the updates in step l:

Updates for β (b and s) For β one notes

$$
\begin{aligned}
&\log(q(b_j, s_j)) \\
&= -\mathbb{E}\frac{\tau}{2}\mathbb{E}_{-j}||y - \mathbf{X}(b \odot s)||_2^2 + \mathbb{E}\log\frac{\pi_{g(j)}}{1-\pi_{g(j)}}s_j - \frac{\mathbb{E}\gamma_{g(j)}}{2}b_j^2 + \text{const} \\
&= -\mathbb{E}\frac{\tau}{2}\left(b_j s_j \sum_k \left(-2y_k\mathbf{X}_{kj} + 2\sum_{l\neq j}\mathbf{X}_{kl}\mathbf{X}_{kj}\mathbb{E}(s_l b_l)\right) + s_j b_j^2 \sum_k \mathbf{X}_{kj}^2\right) \\
&\qquad + \mathbb{E}\log\frac{\pi_{g(j)}}{1-\pi_{g(j)}}s_j - \frac{\mathbb{E}\gamma_{g(j)}}{2}b_j^2 + \text{const.}
\end{aligned}
$$

This can be written as

$$
q(b_j, s_j) = q(s_j = 0)q(b_j|s_j = 0) + q(s_j = 1)q(b_j|s_j = 1),
$$

where

$$
\begin{aligned}
b_j|s_j = 0 &\sim \mathrm{N}(0, (\mathbb{E}\gamma_{g(j)})^{-1}), \\
b_j|s_j = 1 &\sim \mathrm{N}(\mu_j^{(l)}, \sigma_j^{(l)2}),
\end{aligned}
$$

with

$$
\begin{aligned}
\sigma_j^{(l)2} &= (\mathbb{E}\tau||\mathbf{X}_{\cdot,j}||_2^2 + \mathbb{E}\gamma_{g(j)})^{-1}, \\
\mu_j^{(l)} &= \sigma_j^{(l)2}\mathbb{E}\tau\left(-\sum_{k=1}^n\sum_{l\neq j}^p \mathbf{X}_{kj}\mathbf{X}_{kl}\mathbb{E}(\beta_l) + \mathbf{X}_{\cdot,j}^T y\right).
\end{aligned}
$$

To make this scale linearly in p in the inner loop we follow Carbonetto *and others* (2012) and keep track of $v = \mathbf{X}\mu$ and update this only in the new component $v \leftarrow v + (\mu_j^{(\text{new})} - \mu_j)\mathbf{X}_{\cdot,j}$.

The marginal distribution of s_j is given by $s_j \sim \mathrm{Ber}(\psi_j^{(l)})$ with $\psi_j^{(l)}$ obtained from

$$
\begin{aligned}
\operatorname{logit}(\psi_j^{(l)}) &= \mathbb{E}\log\frac{\pi_{g(j)}}{1-\pi_{g(j)}} - \frac{1}{2}\log(\mathbb{E}\tau||\mathbf{X}_{\cdot,j}||_2^2 + \mathbb{E}\gamma_{g(j)}) + \frac{1}{2}\log(\mathbb{E}\gamma_{g(j)}) \\
&\quad + \frac{(\mathbb{E}\tau)^2\left(\mathbf{X}_{\cdot,j}^T y - \sum_{k=1}^n\sum_{l\neq j}^p \mathbf{X}_{kj}\mathbf{X}_{kl}\mathbb{E}(b_l s_l)\right)^2}{2(\mathbb{E}\tau||\mathbf{X}_{\cdot,j}||_2^2 + \mathbb{E}\gamma_{g(j)})^{-1}} \\
&= \mathbb{E}\log\frac{\pi_{g(j)}}{1-\pi_{g(j)}} + \frac{1}{2}\log(\mathbb{E}\gamma_{g(j)}) + \frac{1}{2}\log(\sigma_j^2) + \frac{1}{2}\frac{\mu_j^2}{\sigma_j^2}.
\end{aligned}
$$

This is derived by integrating the joint density of $q(b_j, s_j)$ to obtain the marginal density of s_j. Denoting the normal density with $\varphi(\cdot; \mu, \sigma^2)$ we have

$$
\begin{aligned}
q(s_j) &= \int q(b_j, s_j) db_j \\
&\propto \exp\left(\mathbb{E}\log\frac{\pi_{g(j)}}{1-\pi_{g(j)}} s_j\right) \\
&\quad \int \exp\left(-\mathbb{E}\frac{\tau}{2}\mathbb{E}_{-j}||y - \mathbf{X}(b \odot s)||_2^2 - \frac{\mathbb{E}\gamma_{g(j)}}{2} b_j^2\right) db_j \\
&\propto \exp\left(\mathbb{E}\log\frac{\pi_{g(j)}}{1-\pi_{g(j)}} s_j\right) \\
&\quad \int \varphi(b_j; \mu_j(s_j), \sigma_j^2(s_j))\sqrt{2\pi\sigma_j^2(s_j)} \exp\left(\frac{\mu_j(s_j)^2}{2\sigma_j^2(s_j)}\right) db_j \\
&\propto \exp\left(\mathbb{E}\log\frac{\pi_{g(j)}}{1-\pi_{g(j)}} s_j\right) \sqrt{\sigma_j^2(s_j)} \exp\left(\frac{\mu_j(s_j)^2}{2\sigma_j^2(s_j)}\right) \cdot 1 \\
&= \exp\left(\mathbb{E}\log\frac{\pi_{g(j)}}{1-\pi_{g(j)}} s_j + \frac{1}{2}\log\sigma_j^2(s_j) + \frac{\mu_j(s_j)^2}{2\sigma_j^2(s_j)}\right).
\end{aligned}
$$

Hence,

$$
\begin{aligned}
\log q(s_j) &= \text{const} + \mathbb{E}\log\frac{\pi_{g(j)}}{1-\pi_{g(j)}} s_j + \frac{1}{2}\log\sigma_j^2(s_j) + \frac{\mu_j(s_j)^2}{2\sigma_j^2(s_j)} \\
&= \text{const} + s_j\mathbb{E}\log\frac{\pi_{g(j)}}{1-\pi_{g(j)}} - \frac{1}{2}\log(s_j\mathbb{E}\tau||\mathbf{X}_{\cdot,j}||_2^2 + \mathbb{E}\gamma_{g(j)}) \\
&\quad + \frac{s_j^2(\mathbb{E}\tau)^2\left(\mathbf{X}_{\cdot,j}^T y - \sum_{k=1}^n\sum_{l\neq j}^p \mathbf{X}_{kj}\mathbf{X}_{kl}\mathbb{E}(b_l s_l)\right)^2}{2(s_j\mathbb{E}\tau||\mathbf{X}_{\cdot,j}||_2^2 + \mathbb{E}\gamma_{g(j)})^{-1}} \\
&= \text{const} + s_j\left\{\mathbb{E}\log\frac{\pi_{g(j)}}{1-\pi_{g(j)}} - \frac{1}{2}\log(\mathbb{E}\tau||\mathbf{X}_{\cdot,j}||_2^2 + \mathbb{E}\gamma_{g(j)})\right. \\
&\quad \left. + \frac{1}{2}\log(\mathbb{E}\gamma_{g(j)}) + \frac{(\mathbb{E}\tau)^2\left(\mathbf{X}_{\cdot,j}^T y - \sum_{k=1}^n\sum_{l\neq j}^p \mathbf{X}_{kj}\mathbf{X}_{kl}\mathbb{E}(b_l s_l)\right)^2}{2(\mathbb{E}\tau||\mathbf{X}_{\cdot,j}||_2^2 + \mathbb{E}\gamma_{g(j)})^{-1}}\right\}.
\end{aligned}
$$

In the last steps note $s \in \{0, 1\}$. Comparing this to $s \sim \text{Ber}(\psi)$ where $\log(q(s)) = \text{const} + s\,\text{logit}(\psi)$ we get the above formula for $\psi^{(l)}$.

Taken together, $\beta_j = s_j b_j \sim \delta_0(1 - \psi_j^{(l)}) + \psi_j^{(l)}\text{N}(\mu_j^{(l)}, \sigma_j^{(l)2})$.

Updates for $\gamma = (\gamma_1, \dots, \gamma_G)$

$$\begin{aligned}\log q(\gamma) &= \text{const} + \sum_{j=1}^{p} \left\{ \frac{1}{2} \log(\gamma_{g(j)}) - \frac{\gamma_{g(j)}}{2} \mathbb{E} b_j^2 \right\} \\ &\quad + \sum_{k=1}^{G} \{(r_\gamma - 1) \log(\gamma_k) - d_\gamma \gamma_k\} \\ &= \text{const} + \sum_{k=1}^{G} \left\{ \log(\gamma_k)(r_\gamma - 1 + \frac{1}{2}|\mathcal{G}_k|) - \gamma_k(d_\gamma + \frac{1}{2} \sum_{j \in \mathcal{G}_k} \mathbb{E} b_j^2) \right\}\end{aligned}$$

Thus, $\gamma_k \sim \Gamma(\alpha_k^{\gamma,(l)}, \beta_k^{\gamma,(l)})$ are independent gamma distributions with parameters in step l given by

$$\begin{aligned}\alpha_k^{\gamma,(l)} &= r_\gamma + \frac{1}{2}|\mathcal{G}_k|, \\ \beta_k^{\gamma,(l)} &= d_\gamma + \frac{1}{2} \sum_{j \in \mathcal{G}_k} \mathbb{E} b_j^2.\end{aligned}$$

Updates for τ

$$\log q(\tau) = \text{const} + \frac{n}{2} \log(\tau) - \frac{\tau}{2} \mathbb{E}||y - \mathbf{X}\beta||_2^2 + (r_\tau - 1) \log(\tau) - d_\tau \tau$$

Thus, $\tau \sim \Gamma(\alpha^{\tau,(l)}, \beta^{\tau,(l)})$ is a gamma distribution with parameters in step l given by

$$\begin{aligned}\alpha^{\tau,(l)} &= r_\tau + \frac{n}{2}, \\ \beta^{\tau,(l)} &= d_\tau + \frac{1}{2} \mathbb{E}\beta||y - \mathbf{X}\beta||_2^2.\end{aligned}$$

Updates for $\pi = (\pi_1, \dots, \pi_G)$

$$\begin{aligned}\log q(\pi) &= \text{const} + \sum_{j=1}^{p} \log(\pi_{g(j)}) \mathbb{E} s_j + \log(1 - \pi_{g(j)})(1 - \mathbb{E} s_j) \\ &\quad + \sum_{k=1}^{G} \{(d_\pi - 1) \log(\pi_k) + (r_\pi - 1) \log(1 - \pi_k) - \log(B(d_\pi, r_\pi)\} \\ &= \sum_{k=1}^{G} \log(\pi_k)(d_\pi - 1 + \sum_{j \in \mathcal{G}_k} \mathbb{E} s_j) + \log(1 - \pi_k)(r_\pi - 1 + \sum_{j \in \mathcal{G}_k} 1 - \mathbb{E} s_j)\end{aligned}$$

Thus, $\pi_k \sim \text{Beta}(\alpha_k^{\pi,(l)}, \beta_k^{\pi,(l)})$ are independent beta distributions with parameters in step l given by

$$\alpha_k^{\pi,(l)} = d_\pi + \sum_{j \in \mathcal{G}_k} \mathbb{E}s_j,$$
$$\beta_k^{\pi,(l)} = r_\pi + \sum_{j \in \mathcal{G}_k} (1 - \mathbb{E}s_j).$$

Expected values required The updates above involve the calculation of expected values under the current variational distribution q. These are given by

$$\mathbb{E}\tau = \frac{\alpha^\tau}{\beta^\tau},$$
$$\mathbb{E}\gamma_k = \frac{\alpha_k^\gamma}{\beta_k^\gamma},$$
$$\mathbb{E}\log\frac{\pi_k}{1-\pi_k} = \psi(\alpha_k^\pi) - \psi(\beta_k^\pi),$$
$$\mathbb{E}s_j = \psi_j,$$
$$\mathbb{E}||y - \mathbf{X}\beta||_2^2 = y^T y - 2y^T\mathbf{X}\mu_\beta + \sum_{i,j}(\mathbf{X}^T\mathbf{X})_{i,j}(\Sigma_{i,j}^\beta + \mu_i^\beta\mu_j^\beta),$$
$$\mathbb{E}b_j = \psi_j\mu_j,$$
$$\mathbb{E}b_j^2 = (1-\psi_j)\left(\mathbb{E}\gamma_{g(j)}^{-1}\right) + \psi_j\left(\mu_j^2 + \sigma_j^2\right),$$
$$\mathbb{E}\beta_j = \mathbb{E}b_j s_j = \mu_j\psi_j,$$
$$\mathbb{E}\beta_j^2 = \mathbb{E}b_j^2 s_j = (\mu_j^2 + \sigma_j^2)\psi_j.$$

Here, ψ denotes the digamma function $\psi(x) = \frac{\Gamma'(x)}{\Gamma(x)}$ and

$$\mu^\beta = (\mathbb{E}\beta_j)_{j=1,\dots,p} = (\mathbb{E}b_j s_j)_{j=1,\dots,p},$$
$$\Sigma^\beta = \text{diag}(\text{Var}(\beta_j)_{j=1,\dots,p}) = \text{diag}((\mathbb{E}\beta_j^2 - (\mathbb{E}\beta_j)^2)_{j=1,\dots,p}).$$

Note that here and in the following we dropped the step index (l) of all parameters from the notation for simplicity.

Calculation of the Evidence Lower Bound The evidence lower bound bounds the log model evidence from below and can be calculated in each step to monitor convergence. Recall

$$\log(y) = \mathcal{L}(q) + D_{\mathrm{KL}}(q\,||\,p)$$

with

$$\begin{aligned}
\mathcal{L}(q) &= \mathbb{E}_q \left(\log \frac{p(y, b, s, \gamma, \pi, \tau)}{q(b, s, \gamma, \pi, \tau)} \right) \\
&= \mathbb{E}_q \left(\log p(y, b, s, \gamma, \pi, \tau) \right) + H(q(b, s, \gamma, \pi, \tau)) \\
&= \mathbb{E}_q \left(\log p(y, b, s, \gamma, \pi, \tau) \right) + \sum_{j=1}^{p} H(q(b_j, s_j)) \\
&\quad + H(q(\gamma)) + H(q(\pi)) + H(q(\tau)),
\end{aligned}$$

where $H(q) = \int -q(\theta) \log q(\theta) d\theta$ denotes the differential entropy. The terms from the joint model density are given by

$$\begin{aligned}
\mathbb{E}_q \log p(y, b, s, \gamma, \tau) = \mathbb{E}_q \log p(y|b, s, \tau) + \mathbb{E}_q \log p(b|\gamma) + \mathbb{E}_q \log p(s|\pi) \\
+ \mathbb{E}_q \log p(\gamma) + \mathbb{E}_q \log p(\pi) + \mathbb{E}_q \log p(\tau)
\end{aligned}$$

with

$$\begin{aligned}
\mathbb{E}_q \log p(y|\beta, \tau) &= \frac{n}{2} \mathbb{E} \log(\tau) - \frac{1}{2} \mathbb{E}\tau ||y - \mathbf{X}(b \odot s)||_2^2 - \frac{n}{2} \log(2\pi), \\
\mathbb{E}_q \log p(b|\gamma) &= \sum_j \left(\frac{1}{2} \mathbb{E} \log(\gamma_{g(j)}) - \frac{1}{2} \mathbb{E}\gamma_{g(j)} b_j^2 - \frac{1}{2} \log(2\pi) \right), \\
\mathbb{E}_q \log p(s|\pi) &= \sum_j \left(\mathbb{E}s_j \log(\pi_{g(j)}) + \mathbb{E}(1 - s_j) \log(1 - \pi_{g(j)}) \right), \\
\mathbb{E}_q \log p(\gamma) &= \sum_k \left((r_\gamma - 1) \mathbb{E} \log(\gamma_k) - d_\gamma \mathbb{E}\gamma_k - \log(\Gamma((r_\gamma)) + r_\gamma \log(d_\gamma) \right), \\
\mathbb{E}_q \log p(\pi) &= \sum_k \left((d_\pi - 1) \mathbb{E} \log(\pi_k) + (r_\pi - 1) \mathbb{E} \log(1 - \pi_k) - \log \mathrm{B}(d_\pi, r_\pi) \right), \\
\mathbb{E}_q \log p(\tau) &= (r_\tau - 1) \mathbb{E} \log(\tau) - d_\tau \mathbb{E}\tau - \log(\Gamma((r_\tau)) + r_\gamma \log(d_\tau).
\end{aligned}$$

Here, $B(a, b) = \frac{\Gamma(a)\Gamma(b)}{\Gamma(a+b)}$ denoted the beta function. The required expectations in addition to those used in the updates are easily obtained using the known distributions and parameters of the

variational density in each iteration and the fact that q factorizes, i.e.

$$\mathbb{E}\log(\tau) = \psi(\alpha_\tau) - \log(\beta_\tau),$$

$$\mathbb{E}\tau||y - \mathbf{X}(b \odot s)||_2^2 = \mathbb{E}\tau\mathbb{E}||y - \mathbf{X}(b \odot s)||_2^2,$$

$$\mathbb{E}\log(\gamma_k) = \psi(\alpha_k^\gamma) - \log(\beta_k^\gamma)$$

$$\mathbb{E}\gamma_{g(j)}\beta_j^2 = \mathbb{E}\gamma_{g(j)}\mathbb{E}\beta_j^2,$$

$$\mathbb{E}\log(\pi_k) = \psi(\alpha_k^\pi) - \psi(\alpha_k^\pi + \beta_k^\pi),$$

$$\mathbb{E}(1 - \log(\pi_k)) = \psi(\beta_k^\pi) - \psi(\alpha_k^\pi + \beta_k^\pi).$$

The entropies are derived from the known expression for the entropy of the gamma, beta, Bernoulli and normal distribution, i.e.

$$\begin{aligned}
H(q(b_j, s_j)) &= H(q(b_j|s_j)) + H(q(s_j)) \\
H(q(b_j|s_j)) &= \frac{1}{2}(\log(2\pi) + 1) - \frac{1}{2}\log(s_j\mathbb{E}\tau||\mathbf{X}_{\cdot,i}||_2^2 + \gamma_{g(j)}) \\
H(q(s_j)) &= -(1 - \psi_j)\log(1 - \psi_j) - \psi_j\log(\psi_j) \\
H(q(\gamma)) &= \sum_k (\alpha_k^\gamma - \log(\beta_k^\gamma) + \log(\Gamma(\alpha_k^\gamma)) + (1 - \alpha_k^\gamma)\psi(\alpha_k^\gamma)) \\
H(q(\pi)) &= \sum_k (\log \mathrm{B}(\alpha_k^\pi, \beta_k^\pi) - (\alpha_k^\pi - 1)\psi(\alpha_k^\pi) - (\beta_k^\pi - 1)\psi(\beta_k^\pi) \\
&\quad + (\alpha_k^\pi + \beta_k^\pi - 2)\psi(\alpha_k^\pi + \beta_k^\pi)) \\
H(q(\tau)) &= \alpha^\tau - \log(\beta^\tau) + \log(\Gamma(\alpha^\tau)) + (1 - \alpha^\tau)\psi(\alpha^\tau).
\end{aligned}$$

Multivariate mean-field approximation for β The assumption that the variational distribution $q(\beta)$ factorizes across all predictors can be very strong. Therefore, a more accurate approximation of the true posterior can be obtained by allowing for a p-variate distribution for β.

For $s = 1$, i.e. no spike term in the model, and hence $\beta = b$ the joint distribution in the updates is then given by

$$\log q(\beta) = \text{const} - \frac{\mathbb{E}(\tau)}{2}||y - \mathbf{X}\beta||_2^2 + \sum_{j=1}^p \left\{-\frac{\mathbb{E}(\gamma_{g(j)})}{2}\beta_j^2\right\}$$

Thus, $\beta \sim \mathrm{N}(\mu^{(l)}, \Sigma^{(l)})$ is a normal distribution with parameters

$$\mu^{(l)} = \mathbb{E}(\tau)\Sigma^{(l)}\mathbf{X}^T y,$$

$$\Sigma^{(l)} = (\mathbb{E}(\tau)\mathbf{X}^T\mathbf{X} + D)^{-1} \quad \text{with } D = \mathrm{diag}((\mathbb{E}(\gamma_{g(j)}))_{j=1,\dots,p}).$$

The other updates stay the same, where the covariance matrix Σ is now no longer diagonal as previously. As this update requires the inversion of a $p \times p$ matrix a limiting factor for applying the multivariate mean-field approximation is its computational complexity. When n is small compared to p a better solution is to employ the Woodbury-Matrix identity (Woodbury, 1950), i.e.

$$\Sigma^{(l)} = D - D\mathbf{X}^T((\mathbb{E}(\tau))^{-1}\mathbb{1}_n + \mathbf{X}D\mathbf{X}^T)^{-1}\mathbf{X}D,$$

which requires the inversion of a $n \times n$ matrix only. This multivariate assumption can be useful in the presence of strong correlations between the predictors. In the case where $\mathbf{X}^T\mathbf{X}$ is diagonal we obtain a similar form than for a fully factorized variational distribution.

The evidence lower bound is obtained analogous to the fully factorized case with a multivariate normal distribution and dropping the terms involving s and π. In particular

$$\mathcal{L}(q) = \mathbb{E}_q\left(\log p(y, \beta, \gamma, \tau)\right) + H(q(\beta)) + H(q(\gamma)) + H(q(\tau)),$$

with

$$H(q(\beta)) = \frac{p}{2}(\log(2\pi) + 1) + \frac{1}{2}\log(|\Sigma|).$$

2.2 *Logistic regression model*

In order to adapt the model to binary data, we change the likelihood of y to a Bernoulli distribution and consider a generalized linear model with logistic link function, i.e.

$$y_i|\beta \sim \mathrm{Ber}(\sigma(x_i^T\beta)) \quad \text{with} \quad \sigma(z) = \frac{1}{1 + \exp(-z)}.$$

The priors on the model coefficients β remain the same as in the linear model, the noise variance τ is dropped from the model. While the model is strongly related to the case of the normal response variable, the challenge here lies in the fact that with the Bernoulli distribution for Y we loose the conjugacy of the prior from the linear model. To solve this and still obtain a fast and explicit inference scheme, we use an approximation of the sigmoid function by an exponential of a quadratic term, thus restoring conjugacy.

As $\sigma(-a) = 1 - \sigma(a)$ we can write

$$\begin{aligned}\mathbb{P}(y_i = 1|\beta) &= \sigma(x_i^T\beta),\\ \mathbb{P}(y_i = 0|\beta) &= \sigma(-x_i^T\beta),\end{aligned}$$

and hence the likelihood is given by

$$p(y_i|\beta) = \sigma((2y_i - 1)x_i^T\beta).$$

Following Jaakkola and Jordan (2000) we use the following lower bound on the sigmoid

$$\sigma(z) \geqslant \sigma(\xi)\exp\left(\frac{1}{2}(z-\xi) - \eta(\xi)(z^2 - \xi^2)\right), \qquad \eta(\xi) = \frac{1}{2\xi}\left(\sigma(\xi) - \frac{1}{2}\right).$$

This introduces an additional variational parameter ξ, which we update alongside the other updates to improve this approximation in each iteration.

Using this approximation we can bound the joint density of the model by

$$\begin{aligned}p(y, \beta, \gamma) &= p(y|\beta)p(\beta|\gamma, \pi)p(\gamma)p(\pi)\\ &\geqslant h(\beta, \xi)p(\beta|\gamma, \pi)p(\gamma)p(\pi),\end{aligned}$$

with

$$\begin{aligned}\log h(\beta, \xi) = &\frac{1}{2}\sum_i (2y_i - 1)x_i^T\beta - \sum_i \eta(\xi_i)(x_i^T\beta)^2\\ &+ \sum_i \left(\log(\sigma(\xi_i)) - \frac{1}{2}\xi_i + \eta(\xi_i)\xi_i^2\right).\end{aligned} \tag{2.1}$$

With the fully factorised mean-field assumption we get the following updates:

$$\begin{aligned}
\log(q(b_j, s_j)) &= \text{const} + \log h(\beta, \xi) - \frac{\mathbb{E}(\gamma_{g(j)})}{2} b_j^2 + \mathbb{E} \log \frac{\pi_{g(j)}}{1-\pi_{g(j)}} s_j \\
&= \text{const} + \frac{1}{2} \sum_i (2y_i - 1) x_i^T \beta - \sum_i \eta(\xi_i)(x_i^T \beta)^2 \\
&\quad - \frac{\mathbb{E}(\gamma_{g(j)})}{2} b_j^2 + \mathbb{E} \log \frac{\pi_{g(j)}}{1-\pi_{g(j)}} s_j \\
&= \text{const} + \left(\sum_i (y_i - \frac{1}{2}) \mathbf{X}_{ij} \right) b_j s_j - s_j b_j^2 \sum_{i=1}^n \eta(\xi_i) \mathbf{X}_{ij}^2 \\
&\quad - 2 b_j s_j \sum_{i=1}^n \eta(\xi_i) \sum_{l \neq j} \mathbf{X}_{il} \mathbf{X}_{ij} \mathbb{E}\beta_l - \frac{\mathbb{E}(\gamma_{g(j)})}{2} b_j^2 + \mathbb{E} \log \frac{\pi_{g(j)}}{1-\pi_{g(j)}} s_j .
\end{aligned}$$

Analogous to the linear model we can derive the following updates for the coefficients: $b_j | s_j = 0 \sim \mathrm{N}(0, \mathbb{E}\gamma_{g(j)}^{-1})$ and $b_j | s_j = 1 \sim \mathrm{N}(\mu_j, \sigma_j^2)$ with

$$\begin{aligned}
\sigma_j^2 &= \left(2 \sum_{i=1}^n \eta(\xi_i) \mathbf{X}_{ij}^2 + \mathbb{E}\gamma_{g(j)} \right)^{-1}, \\
\mu_j &= \sigma_j^2 \left(-2 \sum_{i=1}^n \eta(\xi_i) \sum_{l \neq j}^p \mathbf{X}_{ij} \mathbf{X}_{il} \mathbb{E}\beta_l + \mathbf{X}_{\cdot,j}^T (y - \frac{1}{2}) \right).
\end{aligned}$$

The probability for $s_j = 1$ is given by

$$\operatorname{logit}(\psi_j^{(l)}) = \mathbb{E} \log \frac{\pi_{g(j)}}{1-\pi_{g(j)}} - \frac{1}{2} \log(\mathbb{E}\gamma_{g(j)}^{-1}) + \frac{1}{2} \log(\sigma_j^2) + \frac{1}{2} \frac{\mu_j^2}{\sigma_j^2},$$

as in the linear model.

In the case of a multivariate mean-filed assumption on β we obtain

$$\begin{aligned}
\log q(\beta) &= \text{const} + \log h(\beta, \xi) + \sum_{j=1}^p \left\{ - \frac{\mathbb{E}(\gamma_{g(j)})}{2} \beta_j^2 \right\} \\
&= \text{const} + \frac{1}{2} \sum_{i=1}^n (2y_i - 1) x_i^T \beta - \sum_{i=1}^n \eta(\xi_i)(x_i^T \beta)^2 + \sum_{j=1}^p \left\{ - \frac{\mathbb{E}(\gamma_{g(j)})}{2} \beta_j^2 \right\} \\
&= \text{const} + \left(\sum_{i=1}^n (y_i - \frac{1}{2}) x_i^T \right) \beta - \beta^T \left(\sum_{i=1}^n \eta(\xi_i) x_i x_i^T \right) \beta \\
&\quad + \sum_{j=1}^p \left\{ - \frac{\mathbb{E}(\gamma_{g(j)})}{2} \beta_j^2 \right\} .
\end{aligned}$$

Thus, $\beta \sim \mathrm{N}(\mu, \Sigma)$ with parameters

$$\mu = \Sigma \sum_{i=1}^{n} \left(y_i - \frac{1}{2} \right) x_i,$$
$$\Sigma = \left(2 \sum_{i=1}^{n} \{ \eta(\xi_i) x_i x_i^T \} + D \right)^{-1} \quad \text{with } D = \mathrm{diag}((\mathbb{E}\gamma_{g(j)})_{j=1,\dots,p}).$$

Relationship to the linear model Note that the analogy to the linear update becomes explicit, when interpreting Equation (2.1) as a normal density on pseudo-data (Seeger and Bouchard, 2012) defined by

$$\tilde{y}_i = \frac{2y_i - 1}{4\eta(\xi_i)}.$$

Then it can be easily seen that

$$\log h(\beta, \xi) = \log p(\tilde{y}|\beta) + c(\xi),$$

where

$$\tilde{y}_i|\beta \sim \mathrm{N}(x_i^T \beta, (2\eta(\xi_i))^{-1}).$$

Replacing the precision parameter τ in the linear case with the precision of the pseudo-data (which is now sample-specific) can give us above updates directly from the linear model.

Update for the variational parameter ξ The update of the variational parameter ξ is given following Jaakkola and Jordan (2000) by

$$\xi_i^2 = x_i^T (\Sigma + \mu_l \mu_l^T) x_i,$$

which can be restricted to non-negative values of ξ due to the symmetry.

Evidence lower bound As before

$$\mathcal{L}(q) = \mathbb{E}_q \left(\log p(y, b, s, \gamma, \pi) \right) + \sum_{j=1}^{p} H(q(b_j, s_j)) + H(q(\gamma)) + H(q(\pi)).$$

The entropies can be calculated as in the linear model with the respective parameters of the variational distributions. The terms from the joint model density only differ in the first term

$$\begin{aligned}\mathbb{E}_q \log p(y, b, s, \gamma, \pi) = \mathbb{E}_q \log p(y|b, s) + \mathbb{E}_q \log p(b|\gamma) \\ + \mathbb{E}_q \log p(s|\pi) + \mathbb{E}_q \log p(\gamma) + \mathbb{E}_q \log p(\pi),\end{aligned}$$

which here is given by

$$\begin{aligned}\mathbb{E}_q \log p(y|\beta) &= \mathbb{E}_q \log \sigma((2y-1)\mathbf{X}\beta) \\ &\geqslant \mathbb{E}_q \Bigg(\frac{1}{2} \sum_i (2y_i - 1) x_i^T \mu - \sum_i \eta(\xi_i)(x_i^T \beta)^2 \\ &\qquad + \sum_i \left(\log(\sigma(\xi_i)) - \frac{1}{2}\xi_i + \eta(\xi_i)\xi_i^2 \right) \Bigg) \\ &= \frac{1}{2} \sum_i \log(2\eta(\xi_i)) - \frac{1}{2} \sum_i 2\eta(\xi_i)(\tilde{y}_i - x_i^T \mu)^2 + \text{const.}\end{aligned}$$

This provides a lower bound on the evidence lower bound in analogy to the linear model that is used to monitor convergence.

3. Practical considerations

3.1 *Standardization of the predictors*

In penalised regression a common preprocessing step is the standardization of all predictors to unit variance to ensure a presumably 'fair' penalty. This scaling is in 1:1 correspondence to differential penalty factors. Without standardization features on a larger scale would be preferred as they need a smaller coefficient relative to a feature with the same effect but measured on a smaller scale. By default, our method standardizes all features. However, if we want to maintain the difference of variances within each assay, our method can adaptively learn scale differences between assays by γ, thereby removing the need to standardize for adjustment between assays as seen in the CLL application. This could help to retain meaningful differences in the features' variance within one assay as discussed in the CLL application.

3.2 *Modelling an intercept*

To include an intercept into the model, we apply centering of $\mathbf{X}$ and y before model fitting in the case of a linear model. For the logistic model this is not as straightforward and we follow Carbonetto *and others* (2012) in the implementation, i.e. the intercept is β_0 is assumed to have a normal prior $\mathrm{N}(0, \sigma_0^2)$ but considering the limiting case for σ_0 to infinity yielding an improper prior (essentially not penalizing the intercept).

4. Supplementary tables and figures

Algorithm S1 Inference algorithm

1: Input: $\mathbf{X}, y, \bigsqcup_{k=1}^{G} \mathcal{G}_k = \{1, \ldots, p\}$

2: Initialize $\mathbb{E}s_j = 1$, $\mathbb{E}\beta_j$ sampled from $\mathrm{N}(0,1)$, $\mathbb{E}\tau = \mathbb{E}\gamma_k = 1$

3: **while** $\mathcal{L}(q)$ has not converged **do**:

4: **for** $k = 1, \ldots G$ **do**

5: Set $q(\pi_k) = \mathrm{Beta}(\pi_k | \alpha_k^\pi, \beta_k^\pi)$ with

$$\alpha_k^\pi = d_\pi + \sum_{j \in \mathcal{G}_k} \mathbb{E}s_j \text{ and } \beta_k^\pi = r_\pi + \sum_{j \in \mathcal{G}_k} (1 - \mathbb{E}s_j)$$

6: **for** $j = 1, \ldots p$ **do**

7: Set $q(s_j) = \mathrm{Ber}(s_j | \psi_j)$, $q(b_j | s_j = 1) = \mathrm{N}(b_j | \mu_j, \sigma_j^2)$ and

$q(b_j | s_j = 0) = \mathrm{N}(b_j | 0, (\mathbb{E}\gamma_{g(j)})^{-1})$ with

$$\sigma_j^2 = (\mathbb{E}\tau ||\mathbf{X}_{\cdot,j}||_2^2 + \mathbb{E}\gamma_{g(j)})^{-1}$$

$$\mu_j = \sigma_j^2 \mathbb{E}\tau \left(- \sum_{i=1}^{n} \sum_{l \neq j}^{p} \mathbf{X}_{ij} \mathbf{X}_{il} \mathbb{E}(\beta_l) + \mathbf{X}_{\cdot,j}^T y \right)$$

$$\mathrm{logit}(\psi_j) = \mathbb{E} \log \frac{\pi_{g(j)}}{1 - \pi_{g(j)}} + \frac{1}{2} \log(\mathbb{E}\gamma_{g(j)}) + \frac{1}{2} \log(\sigma_j^2) + \frac{1}{2} \frac{\mu_j^2}{\sigma_j^2}$$

8: Set $q(\tau) = \Gamma(\tau | \alpha^\tau, \beta^\tau)$ with

$$\alpha^\tau = r_\tau + \frac{n}{2} \text{ and } \beta^\tau = d_\tau + \frac{1}{2} \mathbb{E}||y - \mathbf{X}\beta||_2^2$$

9: **for** $k = 1, \ldots G$ **do**

10: Set $q(\gamma_k) = \Gamma(\gamma_k | \alpha_k^\gamma, \beta_k^\gamma)$ with

$$\alpha_k^\gamma = r_\gamma + \frac{1}{2} |\mathcal{G}_k| \text{ and } \beta_k^\gamma = d_\gamma + \frac{1}{2} \sum_{j \in \mathcal{G}_k} \mathbb{E}b_j^2$$

11: Calculate $\mathcal{L}(q) = \mathbb{E} \log p(y, b, s, \gamma, \pi, \tau) + H(q)$

Notes: *The expectations are taken under the current variational distribution q, and* $H(q) = \int -q(\theta) \log q(\theta) d\theta$ *denotes the differential entropy. We use* $\mathcal{F}(x|a)$ *to denote the probability density function in x of a distribution* $\mathcal{F}$ *with parameters a, e.g.* $\mathrm{Beta}(x|\alpha, \beta)$. *In step 7 it is important to keep track of* $v = \mathbf{X}\, \mathbb{E}\beta$ *in the implementation to obtain linear computational complexity in p. We set* $r_\tau = r_\gamma = d_\tau = d_\gamma = 0.001$ *and* $d_\pi = r_\pi = 1$.

Table S1. Simulation parameters (p denotes the number of features, n the number of samples, ρ the correlation strength in $\mathbf{X}$, τ the noise precision and ν the sparsity level).

p	n	ρ	τ	ν
60,120,...,1200	100	0	1	0.2
300	20,40,...,500	0	1	0.2
300	100	0, 0.1,...,0.9	1	0.2
300	100	0	0.01,0.1,...,100	0.2
300	100	0	1	0.001,0.01, 0.05, 0.1,...,1

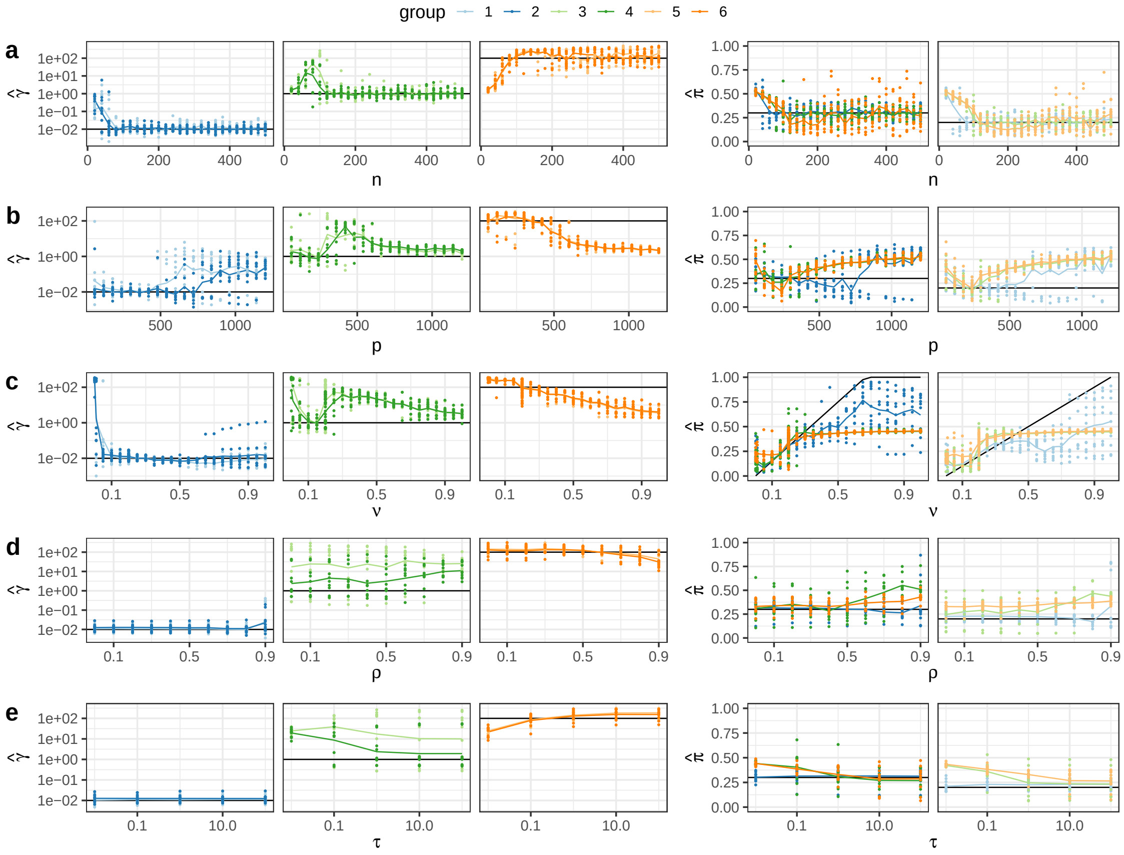

Fig. S1. Estimated values of the hyperparameter γ (left column) and π (right column) when varying each of the model parameters (a-e) while keeping the other four parameters fixed as described in Table S1. The line denotes the mean recovered hyperparameter across 10 random instances of simulated data, while points represent single instances. Colours denote the different groups ($k = 1, \ldots, 6$) and the black line indicates the true value of γ (left) and π (right) used in the simulation. Each panel displays groups with the same value of γ (left) and π (right).

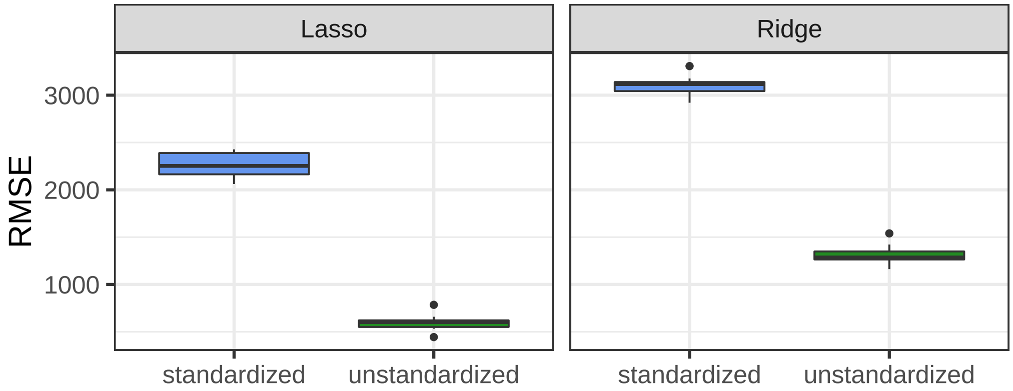

Fig. S2. Simulation example illustrating the effect of standardization in settings with informative high-amplitude features and uninformative low-amplitude features. A number of $p = 600$ features was simulated from a standard normal distribution and multiplied by 10 (high-amplitude features, $p = 300$) or 1 (low-amplitude features, $p = 300$). The response was simulated from a normal model with coefficients given by 1 for the high-amplitude features and 0 otherwise. Lasso and ridge regression were fitted on a training set of $n = 500$ samples using either standardized predictors (blue) or predictors on the original scale (green). The resulting fits were evaluated in terms of the root mean squared error (RMSE) on an independent test set of $n = 500$ samples. The boxplots were obtained from ten independent instances of simulated data.

References

Blei, David M, Kucukelbir, Alp and McAuliffe, Jon D. (2017). Variational inference: A review for statisticians. *Journal of the American Statistical Association* **112**(518), 859–877.

Carbonetto, Peter, Stephens, Matthew *and others*. (2012). Scalable variational inference for Bayesian variable selection in regression, and its accuracy in genetic association studies. *Bayesian analysis* **7**(1), 73–108.

Jaakkola, Tommi S and Jordan, Michael I. (2000). Bayesian parameter estimation via variational methods. *Statistics and Computing* **10**(1), 25–37.

Seeger, Matthias and Bouchard, Guillaume. (2012). Fast variational Bayesian inference for non-conjugate matrix factorization models. In: *Artificial Intelligence and Statistics*. pp. 1012–1018.

Titsias, Michalis K and Lázaro-Gredilla, Miguel. (2011). Spike and slab variational inference for multi-task and multiple kernel learning. In: *Advances in neural information processing systems*. pp. 2339–2347.

Woodbury, Max A. (1950). Inverting modified matrices, memorandum rept. 42. *Statistical Research Group, Princeton University, Princeton, NJ*.
